# Comparison of patients undergoing protected high risk percutaneous coronary intervention using either intravascular lithotripsy or rotational atherectomy

**DOI:** 10.3389/fcvm.2024.1451229

**Published:** 2024-11-29

**Authors:** Tobias T. Krause, Shazia S. Afzal, Anida Gjata, Michael Lindner, Louai Saad, Mirjam Steinbach, Rashad Zayat, Assad Haneya, Nikos Werner, Juergen Leick

**Affiliations:** ^1^ Department of Cardiology, Hospital of the Brothers of Mercy Trier, Trier, Germany; ^2^ Department of Heart Surgery, Hospital of the Brothers of Mercy Trier, Trier, Germany

**Keywords:** high-risk PCI, percutaneous mechanical circulatory support, rotational atherectomy, intravascular lithotripsy, slow reflow

## Abstract

**Background:**

Treating heavily calcified vessels is a challenging task in patients with an impaired left ventricular ejection fraction. Percutaneous mechanical circulatory support (pMCS) is increasingly used in patients in high-risk percutaneous coronary intervention (HRPCI).

**Methods:**

In this retrospective registry, we investigated 25 patients undergoing a protected HRPCI receiving either intravascular lithotripsy (IVL + pMCS; *n* = 11) or rotational atherectomy (RA + pMCS; *n* = 14). The primary endpoint was defined as peri-interventional hemodynamic stability. The secondary endpoint was defined as major adverse cardiac events (MACE).

**Results:**

Patients in the IVL + pMCS group had a significantly higher mean arterial pressure (MAP) at the end of the procedure (*p* = 0.04)*.* However, the Δ-change in MAP was not significant [−12 mmHg (±20.3) vs. −16.1 mmHg (±23.9), *p* = 0.709]. The proportion of patients requiring post-interventional catecholamines was significantly lower in the IVL + pMCS group (*p* = 0.02)*.* The Δ-change in Syntax Score was not significant between groups (IVL + pMCS −22 (±5.8) vs. RA + pMCS −21.2 (±7.6), *p* = 0.783). MACE did occur less in the group of IVL + pMCS (0% vs. 20%, *p* = 0.046). Patients with pMCS insertion as a bailout strategy had a higher probability for in-hospital death (*p* < 0.001) and the occurrence of the slow-reflow phenomenon was associated with long-term mortality (*p* = 0.021) in the cox regression analysis.

**Conclusions:**

In our cohort patients in the IVL + pMCS group were hemodynamically more stable which led to a lower rate of catecholamine usage. pMCS as a bailout strategy was associated with in-hospital death and the occurrence of the slow reflow phenomenon with all-cause mortality during follow-up.

## 1 Introduction

The use of percutaneous mechanical circulatory support (pMCS) systems in the treatment of patients with complex coronary artery disease who are unsuitable for coronary artery bypass surgery due to their clinical presentation, their comorbidities and/or an impaired left ventricular ejection fraction (LVEF) is on the uprise (1). Nonetheless, this population is potentially understudied and 1-year mortality varies from 1% to 11% (2, 3). Improvement in quality of life and a reduction of adverse events can be achieved through coronary intervention in these patients (4–7). With the use of pMCS the operator can perform complex interventions and reduce the risk of a peri-interventional hemodynamical deterioration. This has already been demonstrated in the PROTECT III trial where a significantly lower rate of hemodynamical deterioration, defined as procedural hypotension, cardiopulmonary resuscitation and ventricular arrhythmias occurred (8). However, a universal definition of these high-risk percutaneous coronary interventions (HRPCI) is still lacking (9). Considerations are mainly patient-related factors like criteria of the coronary anatomy and the hemodynamical situation. Also, the patients’ age and past medical history are of interest. Regarding the coronary anatomy, the presence of a coronary multi-vessel disease, last remaining vessel, chronic total occluded coronary arteries and heavily calcified vessels must be considered. Detection of these factors in combination with clinical or hemodynamic signs of cardiac decompensation or an expected long time of myocardial ischemia meets the criteria of a HRPCI (10). Further, clear recommendations of the usage of pMCS in elective cases undergoing HRPCI and especially in patients with severe coronary artery calcification are also lacking. In the presence of calcification, wiring and adequate lesion preparation may be a challenging task and may lead to longer procedure times (11–13). A more intensive lesion preparation carries the risk of complications such as the occurrence of the slow reflow or no reflow phenomenon or coronary artery perforation.

When conventional measures fail in the treatment of severely calcified coronary stenoses, there are alternative methods like intravascular lithotripsy (IVL) or rotational atherectomy (RA) (14). To optimize stent expansion and vessel compliance, IVL, a relatively new therapeutic method for lesion preparation, has been introduced. In this procedure calcium fracturation is achieved by sound wave emitters embedded in a balloon system. In addition, the use of low dilatation pressures (4 atm) reduces barotrauma, which is significantly higher with conventional high-pressure balloon catheters (up to 40 atm). The Disrupt CAD I-IV studies and registries (14–16) demonstrated the benefits and applicability of IVL, as well as the low complication and mortality rates (17). These results and IVL’s safe applicability have been confirmed in further studies (14, 18, 19). However, patients with a severely depressed LVEF were excluded from the Disrupt CAD I-IV studies. The theoretical advantage of RA over IVL is that the calcium is not fractured but ablated by the drill head. The ROTAXUS trial compared RA and stenting with standard therapy and stenting. The patient population had an average LVEF of 55% for the RA group and this trial also did not include patients with pMCS (20). Due to the lack of data in the context of HRPCI with pMCS in patients with severely calcified coronary stenoses, the use of coronary lesion preparation using RA vs. IVL is of interest. Here, with the support of pMCS, the operator can carry out longer procedures with complex interventions with a certain degree of safety, without having to worry about further hemodynamic deterioration of the patient. In this context this is the first study comparing IVL to RA in patients with pMCS.

## 2 Materials and methods

**Figure 1:**
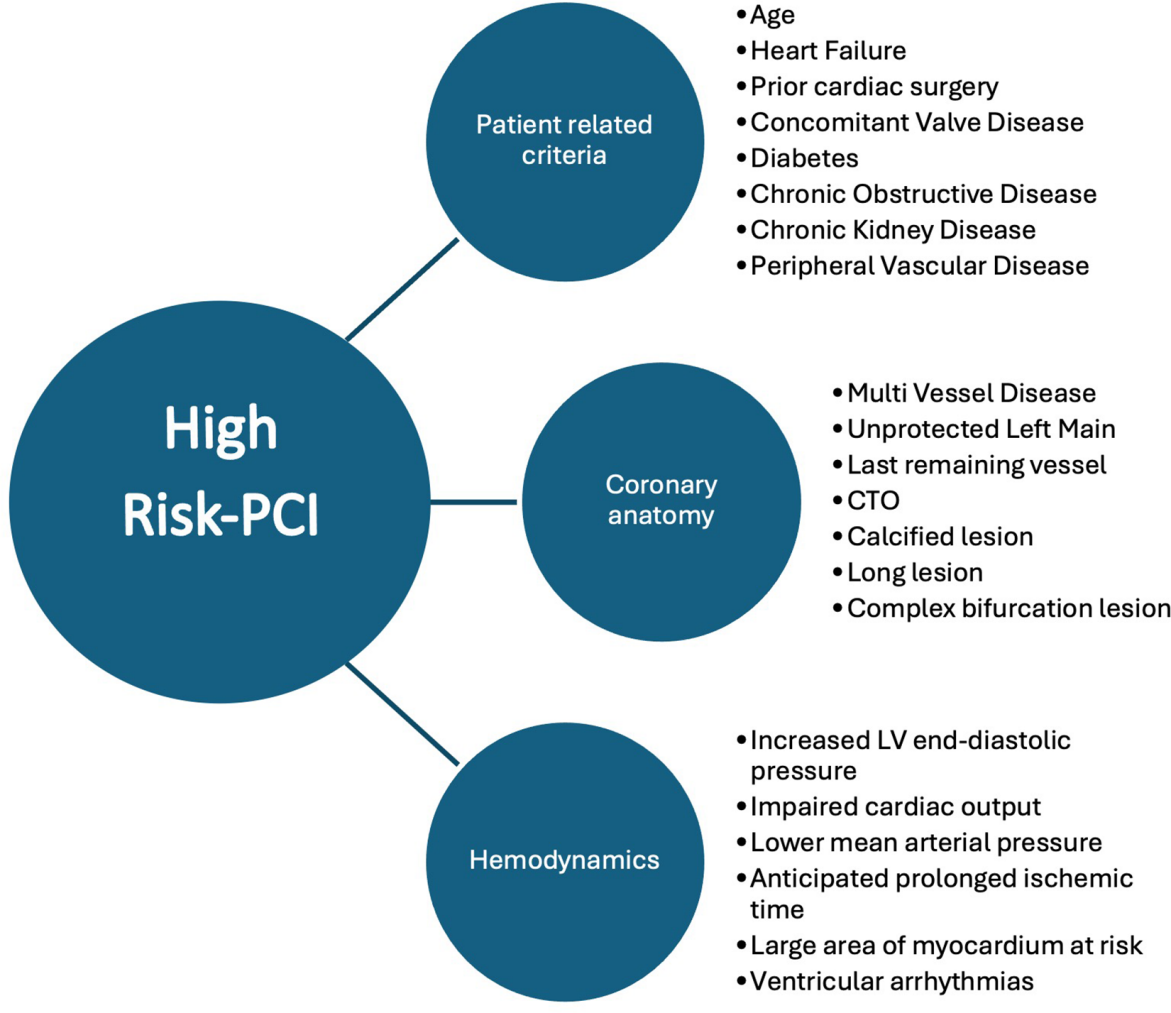
Suggested criteria for defining HRPCI (10).

This retrospective analysis was performed following institutional guidelines and the Declaration of Helsinki of 1975 in its most recent version. Ethical approval of the study was granted by the responsible local ethics committee of the Rhineland-Palatinate chamber of physicians and is listed under the file number 2023-17384. Patient confidentiality has been maintained by anonymizing patient data to remove any identifying information. Thus, patient consent was not required. A local database was generated using standard data collected during hospitalization and during treatment in the catheterization laboratory in the period from September 2019 to July 2023. The data is collected retrospectively from the clinic's internal IT database and is filtered according to the OPS coding for RA (8-837.50), IVL (8-83d.6), and pMCS (8-839.4). From this, patients are identified who received a combination of IVL (Shockwave® Medical Inc. Corporate Headquarters, 5403 Betsy Ross Drive, Santa Clara, CA 95054, USA) and pMCS or RA (Boston Scientific® World Headquarters, 300 Boston Scientific Way Marlborough, MA, USA) and pMCS (Impella® CP SmartAssist®, Abiomed Europe GmbH, Aachen, Deutschland). The use of either IVL or RA was left to the decision of the treating interventional cardiologist. We included all patients with severe coronary artery disease due to complex coronary anatomy, calcified stenoses and corresponding previous illnesses who are not suitable for surgical care. As a standardized approach we based our screening criteria on the recently published criteria suggested for identifying patients suitable for HRPCI (10) (Figure 1). We also included all patients who had an impaired LVEF or one is to be expected to be at risk of hemodynamic compromise during the intervention, based on the characteristics of the lesion. Exclusion criteria consisted of patients age below 18 years, contraindication for pMCS, patients suitable for cardiac surgical treatment, patients receiving Rota-Shock (RA + IVL), and patients in cardiac arrest. Analysis of the lesion characteristics (e.g., length of calcified portion, total length, eccentric, or concentric) was made afterward through a review of the coronary angiography by an operator blinded to the procedure groups. Measurements have been performed retrospectively using offline quantitative coronary angiography (QCA). The primary endpoint was the hemodynamic status after the intervention represented by mean arterial pressure (MAP). The MAP is recorded in our catheterization laboratory as standard before the start and at the end of the procedure. Data were available for all patients. The secondary endpoint was a composite of MACE [cardiac death, stroke, peri-interventional myocardial infarction according to the fourth universal definition of MI (21)] during hospital stay and during the follow-up, which was evaluated by telephone interview.

### 2.1 Statistical analysis

Statistical analysis was performed after collecting all data. Categorical variables are presented as *n* (%). Continuous variables are summarized as mean ± SD or as the median with interquartile range (IQR). For analyzing prevalence χ^2^ test and Fisher's test was calculated. The observations on the achievement of the secondary endpoints were evaluated by survival analyses with determination of univariate and multivariate Cox proportional hazard ratio (HR) and Kaplan–Meier curve. The following variables were included: group, slow reflow, cause of death (cardiac death vs. non-cardiac death) and death overall. For further clarification of predictors of mortality, we investigated the statistical relationship between using pMCS as a bailout procedure and the occurrence of death. Further, we performed multivariate testing for the event of slow reflow in both groups. Lastly, multivariate testing was done for the variable death in combination with the overall event of slow reflow. The analysis was performed with R 4.2.2 (R Foundation for Statistical Computing, Vienna, Austria).

## 3 Results

### 3.1 Baseline and lesion characteristics

In total 25 patients have been enrolled during the screening episode of which 11 patients received IVL and pMCS and 14 patients received RA and pMCS. Use of either calcium modification technique (IVL or RA) for lesion preparation was at the discretion of the interventional cardiologist. Since this was an observational all-comer registry, we especially did not see any difference in patients presenting with acute coronary syndrome and in patients treated in an elective setting in both groups (*p* = 0.999). Further, we observed no differences in baseline characteristics (Table 1), and notably the Syntax Score did not differ between the groups [28.8 (±5.5) vs. 31.9 (±7.7), *p* = 0.255] (Table 2). Lesion characteristics are shown in Table 3. Regarding the group of RA + pMCS, the LAD was the predominantly targeted vessel (*n* = 11, *p* = 0.049). A significantly higher portion of calcification could be found in the RA + pMCS group [13.8 mm (±17.2) vs. 57.1 mm (±18.3), *p* < 0.0001]. This group also had a significantly higher portion of eccentric calcification (*n* = 14 vs. *n* = 7, *p* = 0.026).

**Table 1:** Patient baseline characteristics.

Baseline characteristics	IVL + pMCS (*n* = 11)	RA + pMCS (*n* = 14)	*p*-value
Gender			
Male	8 (32%)	11 (44%)	0.999
Female	3 (12%)	3 (12%)	0.999
Age			
Mean	74.4 (7.9)	76.6 (8.4)	0.493
BMI (kg/m^2^)			
Mean	27.7 (4.8)	27.8 (3.7)	0.94
LVEF (%)			
Mean	42.7 (15.9)	39.8 (16.1)	0.652
Euro score	6.3 (4)	9 (9.5)	0.893
CAD			
CAD 1	0	0	
CAD 2	3 (12%)	2 (8%)	0.623
CAD 3	8 (32%)	12 (48%)	0.623
Cardiovascular risk factors			
Hypertension	10 (40%)	14 (56%)	0.44
Hypercholesterolemia	9 (36%)	10 (40%)	0.661
Diabetes mellitus	5 (20%)	6 (24%)	0.999
Smoking	2 (8%)	1 (4%)	0.565
Positive family history for CAD	2 (8%)	2 (8%)	0.999
NYHA class			
NYHA I	1 (4%)	0	0.44
NYHA II	3 (12%)	3 (12%)	0.999
NYHA III	5 (20%)	7 (28%)	0.999
NYHA IV	1 (4%)	5 (20%)	0.18
CCS class			
CCS I	0	1 (4%)	0.999
CCS II	1 (4%)	2 (8%)	0.999
CCS III	0	4 (16%)	0.105
CCS IV	7 (28%)	6 (24%)	0.428
Acute coronary syndrome	6 (24%)	8 (32%)	0.999
Chronic kidney disease	4 (16%)	5 (20%)	0.999
Impaired renal function	5 (20%)	6 (24%)	0.999
Peripheral artery disease > Fontaine IIb	1 (4%)	3 (12%)	0.604

Absolute prevalence (relative prevalence) for categorical variables. Mean (standard deviation) for metric variables.

**Table 2:** Hemodynamics and procedural data.

Hemodynamics and procedural data	IVL + pMCS (*n* = 11)	RA + pMCS (*n* = 14)	*p*-value
Mean arterial blood pressure (mmHg)			
Pre PCI	99.5 (18)	86.8 (17.4)	0.104
Post PCI	86 (10.5)	73.8 (12.1)	***0***.***04***
Δ Differences of arterial pressure			
Systolic	−19.2 (36.3)	−21.9 (39.1)	0.795
Diastolic	−6.1 (17.1)	−9.4 (15.7)	0.759
MAP	−12 (20.3)	−16.1 (23.9)	0.709
Heart rate (bpm)			
Pre PCI	80.3 (13.8)	72.2 (15.4)	0.207
During PCI	79.9 (17.7)	80.8 (16.5)	0.935
Post PCI	75.2 (11)	82.9 (12.6)	0.218
pMCS as bailout	1 (4%)	5 (20%)	0.18
Catecholamines post PCI	0	6 (24%)	***0***.***02***
Syntax Score			
Syntax Score I pre PCI	28.8 (5.5)	31.9 (7.7)	0.255
Syntax Score I post PCI	6.8 (3.4)	10.6 (5.5)	***0.043***
Δ Syntax Score			
Syntax Score I	−22 (5.8)	−21.2 (7.6)	0.783
Drug eluting stent length (mm)			
Culprit	31.8 (15.8)	69.6 (22.2)	***<0***.***001***
Total	103.8 (36.6)	108.9 (34.5)	0.73
Drug eluting stent diameter (mm)			
Culprit	3.5 (0.6)	3.6 (0.4)	0.436
Minimum	2.7 (0.4)	2.6 (0.2)	0.379
Maximum	3.7 (0.5)	3.8 (0.3)	0.524
Number of stents implanted	4.3 (1.6)	4.4 (1.7)	0.901
Slow reflow	2 (8%)	7 (28%)	0.208
Contrast agent used (ml)	307.9 (102.9)	283.8 (129.2)	0.615
Radiation time (min)	41.7 (10.8)	56.5 (19.4)	***0***.***032***
Procedure time (min)	136.1 (26.6)	182.3 (46.8)	***0***.***001***

Absolute prevalence (relative prevalence) for categorical variables. Mean (standard deviation) for metric variables.

Bold italic values indicate statistical significance with a *p*-value <0.05.

**Table 3:** Lesion characteristics.

Lesion characteristics	IVL + pMCS (*n* = 11)	RA + pMCS (*n* = 14)	*p*-value
Target vessel			
LM	3 (12%)	9 (36%)	0.111
LAD	4 (16%)	11 (44%)	***0***.***049***
LCX	3 (12%)	1 (4%)	0.288
RCA	2 (8%)	1 (4%)	0.565
Lesion length (mm)			
<10	0	0	
10–20	3 (12%)	1 (4%)	0.288
>20	8 (32%)	13 (52%)	0.288
Lesion morphology in treated vessels			
Eccentric	7 (28%)	14 (56%)	***0*.*026***
Concentric	5 (20%)	6 (24%)	0.999
Ostial lesion	6 (24%)	9 (36%)	0.934
De novo lesion	10 (40%)	14 (56%)	0.44
Calcified portion (mm)			
Mean	13.8 (17.2)	57.1 (18.3)	***<0*.*001***
Bifurcation	6 (24%)	7 (28%)	0.999

LM, left main coronary artery; LAD, left anterior descending coronary artery; LCX, left circumflex coronary artery; RCA, right coronary artery.

Absolute prevalence (relative prevalence) for categorical variables. Mean (standard deviation) for metric variables.

Bold italic values indicate statistical significance with a *p*-value <0.05.

### 3.2 Procedural data and primary endpoint analysis

The baseline MAP was slightly higher in the IVL + pMCS group, but without statistical significance (*p* = 0.104). Primary endpoint analysis showed significant higher post-PCI MAP values in the IVL + pMCS group [99.5 mmHg (±18) vs. 86.8 mmHg (±17.4), *p* = 0.04]. However, the ΔMAP change was without statistical significance between the groups [−12 mmHg (±20.3) vs. −16.1 mmHg (±23.9), *p* = 0.709]. The need of catecholamines after PCI was significantly lower in the IVL + pMCS group [*n* = 0 (0%) vs. *n* = 6 (24%), *p* = 0.02].

The calculated baseline Syntax Score and the Δ-difference at the end of procedure did not differ between the groups [−22 (±5.8) vs. −21.2 (±7.6), *p* = 0.783] (Table 2). The length of the drug eluting stent used in the treated culprit lesion was significantly shorter in the group of IVL + pMCS (*p* < 0.001). Procedure and radiation time was significantly lower in the IVL + pMCS group (*p* < 0.001 and *p* < 0.032, respectively).

Detailed information about hemodynamics and procedural data can be found in Table 2.

### 3.3 Secondary endpoint analysis

**Figure 2:**
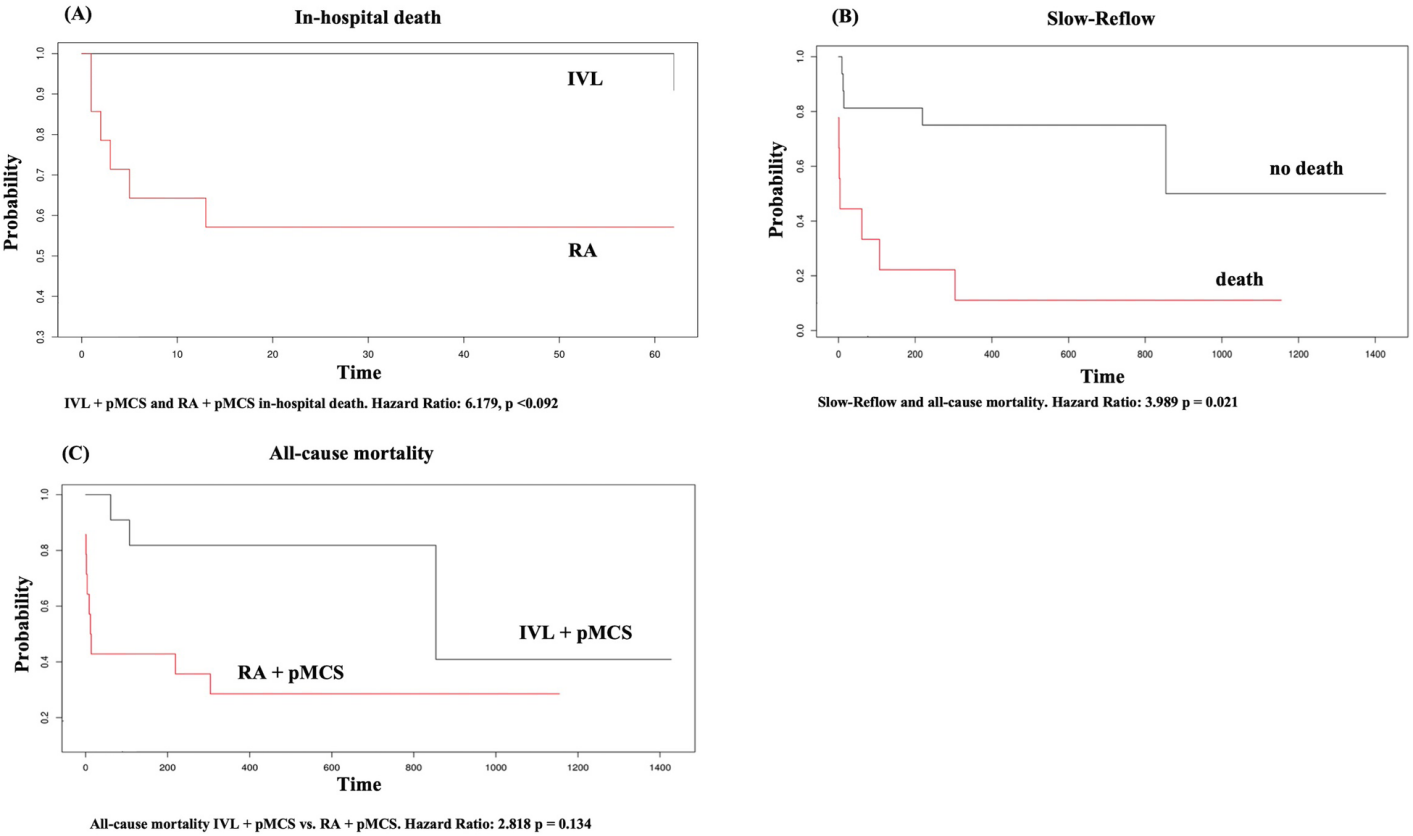
Kaplan Meier plot for **(A)** in-hospital death, **(B)** slow-reflow, **(C)** all-cause mortality. X-axis in **(A–C)** represents time given in days.

We did not find any statistically significant difference for the occurrence of in-hospital death (all-cause mortality) between the groups [*n* = 1 (4%) vs. *n* = 6 (24%), *p* = 0.09] in general. We did find a statistically significant difference for the occurrence of cardiac death (overall) between the groups (*n* = 0 (0%) vs. *n* = 5 (20%), *p* = 0.046). Multivariate regression analysis could exclude an effect of the intervention group on the variable in-hospital death (HR 6.179, 95% CI: 0.741–51.561, *p* = 0.092). However, patients with pMCS insertion as a bailout strategy had a higher probability for in-hospital death in the univariate regression analysis (HR: 51.807, 95% CI: 5.874–456.906, *p* < 0.001). MACE did occur significantly less frequently in the group of IVL + pMCS (0% vs. 20%, *p* = 0.046) and in both group there were no access site bleedings or peripheral leg ischemia reported. Notably, the LVEF at hospital discharge was significantly higher in the group of IVL + pMCS [41.5% (±16.6) vs. 21.7% (±22.4), *p* = 0.035]. During the long-term follow-up [time to survival IVL + pMCS 504.6 days (±377.5) vs. RA + pMCS 278.9 days (±418); *p* = 0.028], we did find a statistically significant lower rate of cardiac death (0% vs. 20%, *p* = 0.046) in the group of IVL + pMCS. Further, all-cause mortality was lower in the IVL + pMCS group (12% vs. 40%, *p* = 0.047). Patients with the slow reflow phenomenon had a higher probability of long-term mortality (HR 3.989, 95% CI: 1.234–12.889, *p* = 0.021). The data on the individual causes of death are shown in Supplementary Table S1. In-hospital and long-term follow-up data are reported in detail in Table 4. The Kaplan–Meier plots for in-hospital death, slow reflow and all-cause mortality can be found in Figure 2.

**Table 4:** In-hospital and long-term follow-up.

Post-procedural data	IVL + pMCS (*n* = 11)	RA + pMCS (*n* = 14)	*p*-value
MACE	0	5 (20%)	***0***.***046***
In-hospital death	1 (4%)	6 (24%)	0.09
All-cause mortality overall	3 (12%)	10 (40%)	***0***.***047***
Cardiac death overall	0 (0%)	5 (20%)	***0***.***046***
Time of survival (days)	504.6 (377.5)	278.9 (418)	***0***.***028***
Stroke	0	0	NA
Acute myocardial infarction	0	1 (4%)	0.999
Access site complications			
Access site bleeding	0	0	NA
Peripheral leg ischemia	0	0	NA
Other access site complications	1 (4%)	4 (16%)	0.105
Packed red blood cells	0	4 (16%)	0.105
LVEF at discharge	41.5 (16.6)	21.7 (22.4)	***0***.***035***
Days to hospital discharge	11.7 (7)	9.6 (2.9)	0.641

Absolute prevalence (relative prevalence) for categorical variables. Mean (standard deviation) for metric variables.

Bold italic values indicate statistical significance with a *p*-value <0.05.

## 4 Discussion

HRPCI procedures are still a challenge, especially in patients with severe calcified coronary arteries. To our best knowledge there are no studies focusing on the comparison of patients receiving protected HRPCI with either IVL or RA so far. Our results can be summarized as follows: first we observed a significantly higher post-PCI MAP in the IVL + pMCS group. However, the ΔMAP change between the groups was not significant. There was no need for catecholamine therapy in the IVL + pMCS group, whereas patients in the RA + pMCS group required catecholamines significantly more often. Second, we observed a significantly lower rate of MACE in the IVL + pMCS group. Third, pMCS as a bailout strategy was associated with in-hospital death and the occurrence of the slow reflow phenomenon was associated with all-cause mortality during the follow-up in the univariate regression analysis.

Regarding the primary endpoint, different factors must be considered. As far as we know there are no randomized studies addressing specifically hemodynamics during IVL in combination with pMCS. Rather, the literature predominantly contains case reports that focus primarily on RA in combination with extracorporeal membrane oxygenation (ECMO) (22, 23). Marchese et al. describe two cases of patients with severe aortic valve stenosis and accompanying severely calcified coronary stenoses that were successfully treated with ECMO and IVL in the left main (24). Dallan et al. were able to describe a successful intervention of RA in the left main using Impella (25). Buono et al. published a case report of a heavily calcified left circumflex in a patient with an impaired LVEF of 25% treated by IVL and Impella (26). A larger group of patients receiving RA + pMCS can be found in the PROTECT III trial (8). It is of note that these were only elective patients and a carefully selected study collective. Furthermore, in the PROTECT III trial there was no subgroup analysis investigating RA with other plaque-modifying procedures. In none of the PROTECT III cases a hemodynamic instability was reported. In our study we observed a significantly higher rate of post-PCI usage of vasopressor in the RA + pMCS group. Although a significantly higher MAP was seen in the IVL + pMCS group during the procedure, the Δ-difference of the MAP in the two groups did not differ in statistically significant terms. We therefore conclude that patients in the RA + pMCS group in our cohort were in a significantly higher need of vasopressors to maintain stable hemodynamics for the same degree of revascularization reflected by the statistically non-significant post-procedural Syntax Score delta change. Notably, although the Syntax Score did not differ before the intervention the above-mentioned findings might indicate that the patient group of RA + pMCS is more complex than the group of IVL + pMCS patients. In addition, IVL has been reported in the ROTA.shock trial as non-inferior to RA regarding minimal stent area (27). However, this complexity seems not to be reflected by the Syntax Score appropriately. One must keep in mind that although not statistically significant the higher amount of slow reflow in the RA + pMCS group and the higher rate of pMCS as a bailout strategy might contribute to the higher rate of catecholamine usage and the higher rate of MACE. Furthermore, for pMCS as a bailout strategy we did find a statistical significance in the probability of in-hospital death in the univariate testing. Although there is no difference in the event of in-hospital death between the groups there is a higher risk of fatal outcome if pMCS is used as a bailout strategy during PCI in cases of hemodynamic instability. This is in line with the findings of Basir et al. who demonstrated an early use of pMCS improves hemodynamics and survival rates in patients with acute myocardial infarction (28). The same was demonstrated in the DanGer Shock study (29). However, these studies treat patients in cardiogenic shock and the data are therefore not comparable with our data. We therefore emphasize the necessity of a standardized patient selection, strategy planning beforehand as well as an early usage of pMCS in the context of HRPCI, also in elective cases. A combination of different characteristics consisting of patient-related criteria (e.g., age, heart failure), the coronary anatomy (e.g., multi-vessel disease, last remaining vessel) and hemodynamics (e.g., increased left ventricular end-diastolic pressure, impaired cardiac output) have been published recently to identify patients at risk (10) (Figure 1).

Addressing the secondary endpoint, a significantly higher MACE rate in the RA + pMCS group was observed. Patients in the RA + pMCS group had longer calcified lesions treated which led to a more intensive lesion preparation, as well as longer procedural duration which might resulted in a numerical but statistical not significant higher rate of slow reflow phenomenon in this group. In addition, during and after the usage of RA thermal injury and platelet activation are reported (30), which might be associated with the increased MACE rate in the RA + pMCS group. However, multivariate testing did not show any difference in the probability of slow reflow between the groups, but when slow reflow occurred, a statistically significant increase in the probability of all-cause mortality was observed. Further, a recent study reports a significant higher 2-year mortality rate in patients with an impaired LVEF (<35%) receiving RA (31). In our cohort, the LVEF change in the RA + pMCS group must be considered as multifactorial and a combination of slow reflow, longer length of calcification and a non-statistically significant higher percentage of pMCS as a bailout strategy in the RA group. The significantly lower LVEF at discharge in the RA + pMCS group might also contribute to the fact that a higher rate of cardiac death (0% vs. 20%, *p* = 0.046) and all-cause mortality (12% vs. 40%, *p* = 0.047) in the long-term follow-up could be seen. Observational studies have demonstrated a nearly two times higher rate of death and electric instability in patients with coronary artery disease and reduced LVEF receiving incomplete revascularization (32, 33). In this context the combined data of the Protect II study and the cVAD registry shows that a more complete revascularization is independently associated with a better LVEF (34).

## 5 Limitations

Several limitations of our study have to be considered. This single center experience study with a retrospective approach and a small number of patients limits the generalizability of the statistical findings. However, the operator driven choice of either using IVL or RA reflects a real-world population but inherits a potential selection bias. Excluding patients who received a combination of RA and IVL further limits the generalizability in especially difficult lesions. Also, only RA and IVL were used as calcium modification methods but other devices like orbital atherectomy were not included due to a lack of routine use in this single center study. Further, some patients were treated in an elective setting, others were admitted during acute coronary syndrome which might influence the overall outcome of the patients, although there was no significant difference in the number of acute coronary syndrome patients in both groups. Intravascular imaging was not standardly used in all cases (16%). Although this rate was even higher than the average rate of intravascular imaging of 6.6% as reported by Lemor et al. in 2020, it was not valid to be used for statistical analysis (35). The choice of calcium modification tool was mostly done angiographically. Use of either calcium modification technique (IVL or RA) for lesion preparation was at the discretion of the interventional cardiologist. As the selection of the calcium modification method was subject to the operator, a possible bias cannot be ruled out. Nevertheless, in future studies systematic intravascular imaging will give a better understanding of plaque morphology and will help identify the best plaque modification device to choose. Despite multivariate testing confounders like degree of calcification and procedural complexity were more prevalent in the RA group and this study was not adequately powered for its secondary endpoint. There was no routine follow-up angiography to identify long term procedural failure (e.g., in stent restenosis).

## 6 Conclusion

In our cohort patients in the IVL + pMCS group were hemodynamical more stable, which led to a lower rate of catecholamine usage. This might be related to our findings of a higher LVEF, a lower MACE rate, a lower overall cardiac death rate and a lower all-cause mortality in patients receiving IVL + pMCS. However, in the multivariate analysis, a correlation between the intervention group and the occurrence of in-hospital death could be ruled out. We did observe a statistical significance between the occurrence of slow reflow and all-cause mortality, whereas the slow reflow phenomenon did not differ statistically between the two groups. In addition, we did observe a significant correlation between the event of in-hospital death if pMCS is used as a bailout strategy in patients qualifying for HRPCI. This emphasizes the need of a common definition for HRPCI to identify patients at risk to be able to perform proper procedure planning and patient preparation. Even more, it substantially underlines the necessity of early pMCS usage if these patients are identified, as already reported by Basir et al. (28). When screening for HRPCI patients, the LVEF is one of the key factors that determines whether to use or not to use pMCS. This is in line with recent studies which included patients with a LVEF <45% and a planned HRPCI with or without pMCS (36, 37). Furthermore, the occurrence of slow reflow during HRPCI should indicate a closer observation of the treated patient as it may trigger a higher all-cause mortality and can be helpful in categorizing patients into HRPCI groups. In light of the limitations of this study, the findings can only be used for hypothesis generation. Larger patient cohorts, standardized patient selection and a standardized procedure protocol with routine follow-up investigations are needed.

## Data Availability

The original contributions presented in the study are included in the article/Supplementary Material, further inquiries can be directed to the corresponding author.
