# Supplementary material for: Comparison of patients undergoing protected high risk percutaneous coronary intervention using either intravascular lithotripsy or rotational atherectomy
Source: Front Cardiovasc Med. 2024 Nov 29;11:1451229. doi: 10.3389/fcvm.2024.1451229 (PMC11638216; doi:10.3389/fcvm.2024.1451229)
Supplement: Supplementary file 1 [file Table1.pdf]

Supplementary Table 1. Individual cause of death

Cause of Death	IVL + pMCS	RA + pMCS
In-hospital	septic shock (n=1)	cardiogenic shock (n=4)
		septic shock (n=1)
		pneumonia (n=1)
Cardiac death overall		cardiogenic shock (n=4),
		acute coronary syndrome (n=1)
All-cause mortality	kidney failure (n=1)	cardiogenic shock (n=4)
	septic shock (n=1)	acute coronary syndrome (n=1)
	multi organ failure (n=1)	pneumonia (n=1)
		ZNS lymphoma (n=1)
		multi organ failure (n=1)
		septic shock (n=1)
		unclear (n=1)
